# Toxoplasmosis and Chlamydophilosis in Small Ruminant Farms in Cameroon: Knowledge, Attitudes, Practices, and Perception of Zoonotic Risks of Farmers

**DOI:** 10.1155/2024/2946764

**Published:** 2024-05-15

**Authors:** Roland Nankam Chimi, Justin Kouamo, Michel Alain Simo Kouam, Muller Dzousse Fotsac, Raphael Chermapi Dembeng, Armelle Prudence Kouengoua Kouengoua, Josué Simo Louokdom, Ferdinand Ngoula

**Affiliations:** ^1^School of Veterinary Medicine, Higher Institute of Health Sciences, Université des Montagnes, P.O. Box 208, Bangangte, Cameroon; ^2^Laboratories of Animal Physiology and Health, Department of Animal Science, Faculty of Agronomy and Agricultural Sciences, University of Dschang, P.O. Box 222, Dschang, Cameroon; ^3^School of Veterinary Medicine and Sciences, The University of Ngaoundere, P.O. Box 454, Ngaoundere, Cameroon; ^4^Pharmacy Division, Higher Institute of Health Sciences, Université des Montagnes, P.O. Box 208, Bangangte, Cameroon

## Abstract

Zoonotic abortive diseases represent a significant health and economic risk for national public health. This cross-sectional survey was conducted from April to October 2021 among 200 selected small ruminant farmers in the three northern regions of Cameroon. Data collection was done through questionnaires administered by exchange with the herder, and responses were coded and recorded on an Excel spreadsheet. The data were then analyzed with R software, version 2.13.0. An ANOVA test was used to assess significant differences in mean of Knowledge, Attitudes, Practices, and Perception of zoonotic risks (KAPP) scores between regions. Pearson correlations were used to calculate the association between KAPP scores within regions. Small ruminant farmers surveyed had low mean scores for knowledge of abortive toxoplasmosis and chlamydophilosis (0.1 ± 0.2), desirable attitude (0.32 ± 0.07), appropriate practice (0.36 ± 0.13) in managing abortion, and positive perception of zoonotic risks of toxoplasmosis and chlamydophilosis in small ruminants (0.12 ± 0.33), respectively. KAPP was significantly (*P* < 0.01) and positively associated with knowledge (*r* = 0.98) and risk perception (*r* = 0.99). However, attitudes (*r* = 0.06), practices (*r* = 0.05), and risk perception of toxoplasmosis (*r* = 0.07) and chlamydophilosis (*r* = 0.08) were not associated with farmers' knowledge. This study revealed significant knowledge gaps, low levels of desired attitudes, and high-risk behavioral practices. These results therefore call for capacity building of health professionals and farmers to better integrate the One Health concept in the management of neglected zoonotic diseases.

## 1. Introduction

Toxoplasmosis and chlamydophilosis are widespread diseases worldwide, particularly in tropical areas, and are currently the leading causes of infectious abortion in small ruminant livestock [1]. The importance of these diseases in terms of frequency, economic losses, and direct and indirect consequences on human health is well documented [2–5]. Nowadays, high abortion rates seriously compromise the cost-effectiveness of sheep and goat farming. In countries such as the United Kingdom (UK), it is estimated that the cost of an abortion in beef sheep can exceed €90. This cost is always much higher in dairy sheep, reaching a minimum of €300 in high-productivity farms. Some countries, such as Canada and Switzerland, consider the abortion rate to be normal if it is between 1% and 5% [6]. In the literature, several factors influence the management of abortion on farms. Studies carried out on the perception of abortion among sheep and goat farmers show that very few call a veterinarian following an abortion [6–8]. This low rate of calls to the vet is explained by the economic argument. In addition, farmers report that they are unaware of the protocol to be followed in the event of an abortion, and few are aware of the zoonotic potential of abortifacient infectious agents in small ruminants. It was also found that the abortion rate was significantly higher (*P* < 0.05) where the number of ewes or goats was only around twenty, and lower on larger farms. This may be explained by the hobbyist nature and low level of knowledge of these farmers.

They are difficult to control on farms and are a complex problem in Cameroon because on the one hand, breeders do not declare abortion cases to the public services, and on the other hand, aborted females usually sell to other breeders or slaughtered for human consumption. Added to this is the management of runts and embryonic annexes, most of which are discarded in the wild and/or consumed by domestic or wild carnivores [2, 9, 10]. In addition, the small ruminant production system in Cameroon is mostly integrated with cattle breeding, which means that some farmers have to transhumance with their small ruminants, making it difficult to control these diseases [11–14]. These diseases are poorly understood by the general population, which is justified by their high prevalence in human hospital structures in Cameroon [15–18]. Breeders and pregnant women, especially those living in rural areas, generally need to be made aware of the risks of abortifacient zoonotic diseases and how to avoid them.

In Cameroon, more than 3/4 of the national small ruminant livestock population comes from the northern regions [19] and *Chlamydophila abortus* and *Toxoplasma gondii* represent the two main causes of abortion in livestock [9, 20]. Therefore, this study was conducted to assess the level of knowledge, attitudes, practices, and perception (KAPP) of zoonotic risk among small ruminant breeders faced with infection/infestation by toxoplasmosis and chlamydophilosis on farms. The data obtained will be used to set up a management plan to raise awareness among farmers and the general public of the risks associated with animal abortion products.

## 2. Materials and Methods

### 2.1. Study Area Description

**Figure 1:**
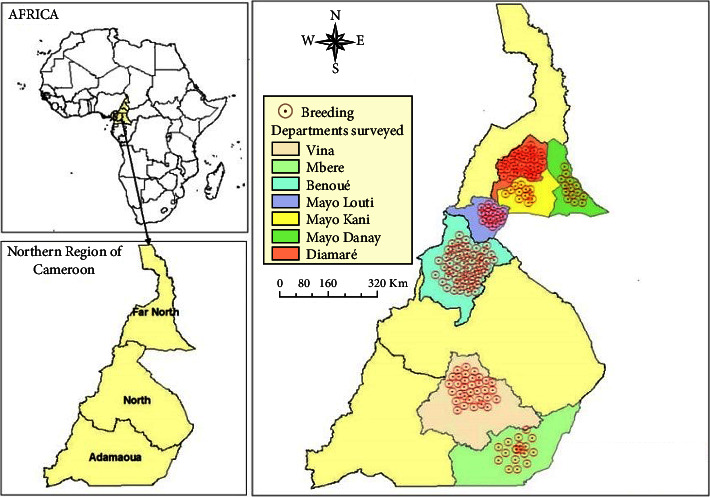
Map showing the study regions (Adamawa, North, and Far North) in Cameroon.

The current investigation was conducted in the northern regions of Cameroon (Adamawa, North, and Far North) (Figure 1) [9]. These regions account for more than 75% of the small ruminant population in Cameroon and are experiencing strong growth in livestock of various species. The Adamawa region covers an area of 64,000 km^2^, making it the third largest region in Cameroon. The land is poor and sparsely populated. The main economic activity is cattle breeding. It lies between 6° 30′ 0″ north latitude and 13° 30′ 0″ east longitude. The region's high altitude results in a relatively cool climate of between 22 and 25 degrees. The North Cameroon region extends between 8° and 10° north latitude and 12° and 16° east longitude, with a surface area of 6,557,600 ha and a population density of 26 inhabitants/km^2^. The climate is hot and semi-arid, with a rainy season from mid-May to September, while the rest of the year is characterized by heat and drought. The Far North region covers an area of 3,4,263 km^2^ and borders Tchad and Nigeria. Its relief is dominated by steppe and grassy savanna, interspersed with strangely shaped massifs. Extreme Nord has a desert climate according to the Köppen–Geiger classification. The surveys were conducted in small ruminant farms that had experienced abortions in the two years preceding the survey.

### 2.2. Study Population Description

Northern Cameroon is home to a complex mix of tribes and peoples. Identities in this region are not fixed, but in flux. The indigenous population is the Mbororo. They are nomadic herders spread across the entire national territory, with heavy concentrations in the northern and southern parts of Cameroon. They are part of a large group that the British called the Fulani or Peul in French. Their main activity is livestock breeding, which is passed down from generation to generation. They are a Fulani people, generally with no formal education. The study was carried out among these small ruminant breeders.

### 2.3. Study Design

A descriptive cross-sectional survey of small ruminant breeders was conducted from April to October 2021 in the northern regions of Cameroon (Adamawa, North, Far North) to assess their level of knowledge, attitudes, practices, and perception (KAPP) of the zoonotic risk related to toxoplasmosis and chlamydophilosis on farms.

### 2.4. Data Collection

Data collection was carried out using questionnaires administered during interviews, and responses were in dichotomous ordinal and categorical form. Moreover, we also had open-ended questions. The questionnaire was administered in French and in the vernacular language for a more fluid survey and to allow the populations of the said regions to feel concerned by the study. Demographic characteristics of small ruminant breeders (sex, age, education, marital status, duration of training in small ruminant breeding, and experience) as farm and animal characteristics (species, sex, breeds, age groups, farm size, number of animals, and level of hygiene) were collected. In addition, the knowledge, attitudes, and practices of small ruminant farmers regarding toxoplasmosis, chlamydophilosis, and risk perception were collected. The questionnaire was tested for comprehension/validity/reliability/language/ease of use on a subset of 10 small ruminant farmers randomly selected from the study regions and adapted accordingly.

### 2.5. Sampling Procedures

The minimum size of 99 farmers was calculated according to the formula proposed by Musallam et al. [21]. This formula proposes to calculate the minimum sample size of the herds and then to calculate the minimum sample size of the animals in the selected herds. Firstly, it takes into account the surface area (1 − *α*) of the normal curve (*Z*) (*Z* = 1.65), the known or attributed prevalence of the two pathologies and the sensitivity and specificity of the ELISA test used within the herd (HSe and HSp = 1) with an absolute precision of 10%. Secondly, it integrates the probability of detecting at least one positive animal and the expected number of infected animals, taking into account the number of herds selected. This minimum size was estimated based on a sero-epidemiological survey of chlamydophilosis and toxoplasmosis in small ruminants in Mali [2]. Small ruminant farms with abortions in the last two years were included in the study. This inclusion criterion resulted in a sample size of 200 small ruminant farmers. The sample was stratified according to the number of farmers in each study region, to ensure a representative geographical coverage. During the survey, the investigators, through technical observations of the farms, characterized the hygiene level of the farms studied on a scale of 1 to 4 [22]. The observations were based on the average score of the different hygiene levels (4 = Very clean, 3 = Clean, 2 = Dirty, and 1 = Very dirty).

### 2.6. Data Analysis

Once the data collection was done, an Excel template was developed to code the completed questionnaires in the study regions and the interpretation was done according to the KAPP survey evaluation grid developed by Essi and Njoya in 2013 [23]. The evaluation of KAPP was done first by totaling the number of points obtained correspondingly in each item of the questionnaire. Each correct answer was worth 1 point for the “knowledge” item, 2 points for the “attitude-practice” item, and 1 point for the “risk perception” item. However, wrong answers were worth 0 points. For questions with several choices, the number of points was obtained by dividing the point corresponding to the question by the number of choices. The results were then converted into a percentage for an overall assessment as presented in the Essi et al. assessment grid. Descriptive statistics using R software were used to indicate the demographic distribution of farmers and farm characteristics. One-way ANOVA associated with post hoc Duncan test analysis was used to assess significant differences of mean KAPP scores across regions. For dichotomous variables, an independent *t*-test was used for comparison. The mean of the scores ± standard deviation was used to represent the level of knowledge, desirable attitudes, appropriate practices, and positive risk perception of toxoplasmosis and chlamydophilosis. Pearson correlations were used to assess the relationship between mean KAPP scores between and within regions.

## 3. Results

### 3.1. Demographic Characteristics of Small Ruminant Farmers in Cameroon

A total of 200 questionnaires were completed during the survey. Most of the respondents included in the study were males (80%), aged 21–50 years (56.5%). About 96.5% of the farms had no animal health staff and 36.5% of the respondents had no education. Most of these farmers were married monogamously (54.5%), and small ruminant rearing was the main activity for 62.6% of them, most of whom (52.5%) had been doing it for 10 to 20 years. Financial income was the main objective for 94.5% of farmers; only 2% practiced this activity for self-consumption and 3.5% for pleasure. The workforce was mainly family-based (98%), with a minority of farms (2%) employing mainly salaried workers.

### 3.2. Characteristics of Small Ruminant Farms Studied in Cameroon

37.50% of small ruminant flocks in the northern regions were made up of sheep and goats, most of which were between 10 and 20 heads (50%). Reproduction has been very often uncontrolled, using natural mating (100%). Feeding was mainly pasture-based (89.5%), with watering mainly from streams (90.5%). Infections of the digestive system (33.7%) and parasitism (20.4%) are frequently encountered on these farms. Less than 1% of farmers used laboratory tests to identify diseases on their farms. As far as abortions were concerned, the farmers interviewed had regularly (96.50%) reported cases of abortion on their farms. The causes of abortion were not investigated for most of those questioned (98.50%), and the stage of gestation at the time of abortion was unknown for 97% of them. However, 91% of those surveyed had not carried out postabortion treatment, and not all had applied prophylactic measures. The majority of abortions recorded on farms were concentrated in one period of the year (48.5%), generally between March and August (84.55%).

### 3.3. Knowledge of Small Ruminant Farmers on Toxoplasmosis and Abortive Chlamydophilosis

**Figure 2:**
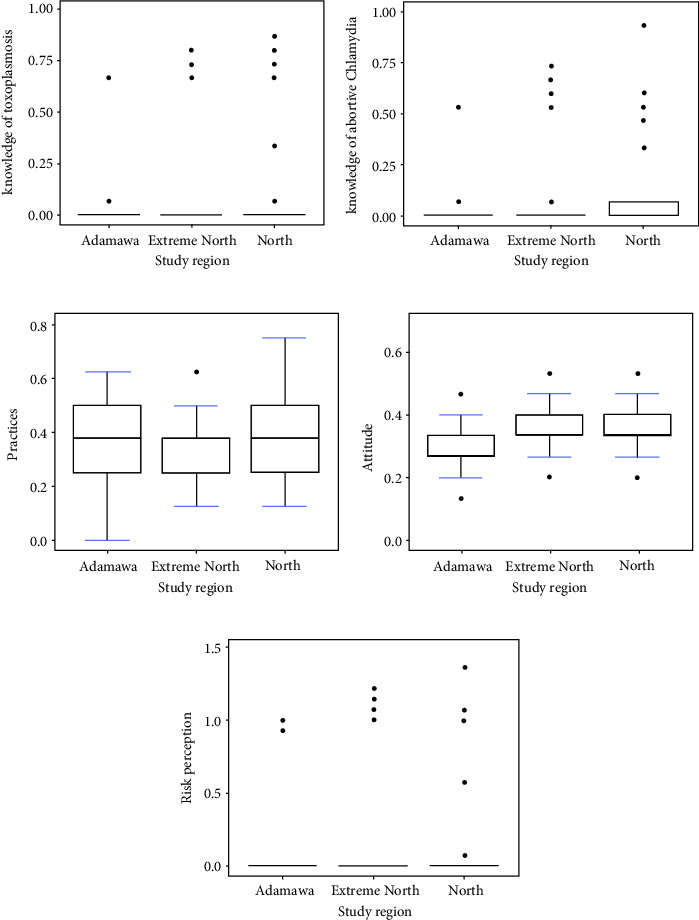
Distribution of mean scores of knowledges of toxoplasmosis (a), chlamydia (b), practices (c), and attitudes (d) toward abortions in small ruminants and risk perception (e) of toxoplasmosis and chlamydia of small ruminant farmers (*N* = 200) in the Adamawa, North, and Far North of Cameroon.

**Figure 3:**
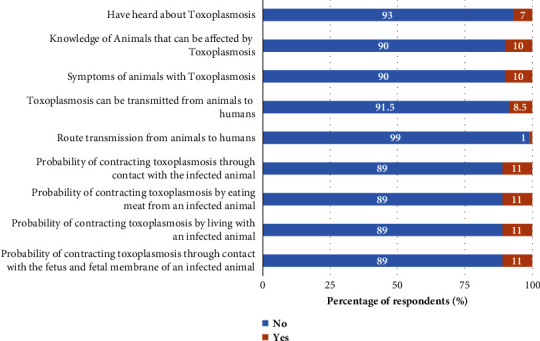
Distribution of small ruminant farmers' knowledge of toxoplasmosis in the Adamawa, North, and Far North regions of Cameroon (*N* = 200).

**Figure 4:**
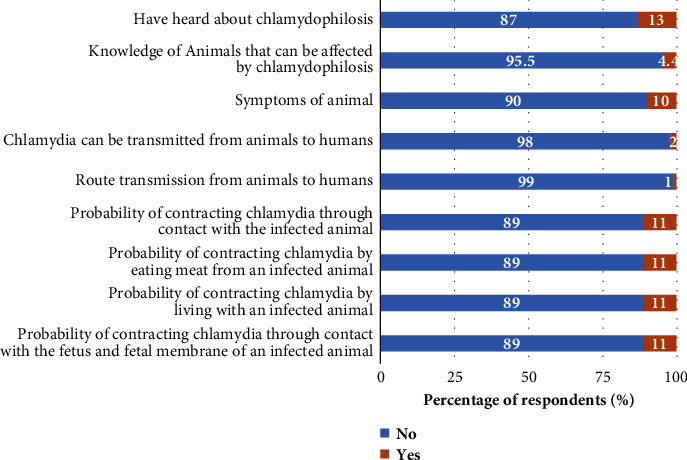
Distribution of small ruminant farmers' knowledge on chlamydophilosis in Adamawa, North, and Far North regions of Cameroon (*N* = 200).

The distribution of small ruminant farmers' knowledge of toxoplasmosis and chlamydophilosis in the surveyed areas is presented in Table 1. Overall, the mean knowledge scores of toxoplasmosis and chlamydophilosis of small ruminant farmers in the study regions were 0.1 ± 0.2. Region, age, marital status, level of education, occupation, purpose of farming, level of hygiene, and feeding method were the main factors influencing knowledge scores. The study also showed that the mean knowledge scores on toxoplasmosis and chlamydophilosis were significantly (*P* < 0.05) higher in the North [(0.12 ± 0.27) and (0.10 ± 0.21)] and Far North regions [(0.07 ± 0.21) and (0.06 ± 0.17)], respectively, than in the Adamawa region (0.03 ± 0.14) (0.03 ± 0.10) (Figures 2(a) and 2(b)). The criteria for assessing the level of knowledge of small ruminant farmers revealed that 10% have heard of these diseases, 8% know which animals can be affected by toxoplasmosis and chlamydophilosis, 10% know the symptoms in animals and can describe them, and 5% know that humans can be infected by these diseases. Associated with this, very few know the different routes of transmission (1%), but 11% say that it is possible to contract these diseases by various means, including living with an infected animal, contact with an infected animal, eating meat from an infected animal, and contact with the fetus and fetal membrane of an infected animal (Figures 3 and 4).

### 3.4. Farmers' Practices and Attitudes toward Small Ruminant Abortions

**Figure 5:**
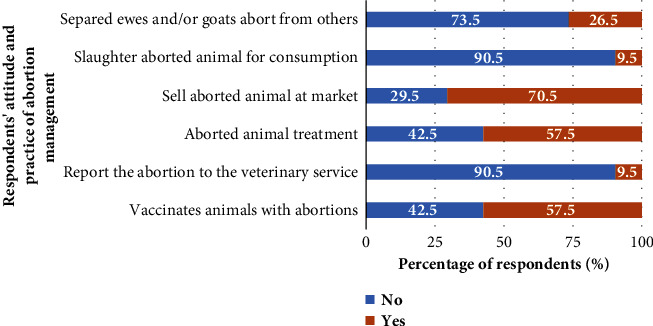
Distribution of abortion management practice and attitude by small ruminant farmers in Adamawa, North, and Far North regions of Cameroon (*N* = 200).

Measure criteria, such as separation of aborted ewes and/or goats from others, slaughter of the aborted animal for consumption, sale of the aborted animal on the market, treatment of the aborted animal, declaration of the abortion to the veterinary service, and vaccination of aborted animals, had been used to assess the practices and attitudes of small ruminant breeders in relation to the management of abortions on the farm (Figure 5). Half of those surveyed had adopted appropriate practices (0.36 ± 0.13), particularly in the Adamawa (0.38 ± 0.14) and northern (0.36 ± 0.14) regions of Cameroon (Figure 2(c)). Regarding farmers' attitudes to abortion, respondents in the Far North (0.33 ± 0.08) and North (0.33 ± 0.07) regions had more appropriate attitudes than those in the Adamawa region (0.29 ± 0.07) (*P*=0.01) (Figure 2(d)). The distribution of mean scores for abortion practices and attitudes of small ruminant breeders on surveyed farms is presented in Table 2.

### 3.5. Perception of Risks on Toxoplasmosis and Chlamydophilosis by Small Ruminants Farmers in Cameroon

**Figure 6:**
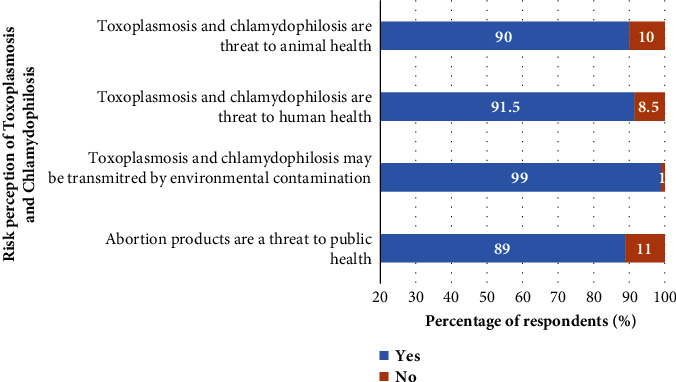
Distribution of risk perception of toxoplasmosis and chlamydophilosis by small ruminant farmers (*N* = 200) in the Adamawa, North, and Far North regions of Cameroon.

As for the perception of risks by the interviewed farmers, a mean score of 0.13 ± 0.38 for toxoplasmosis and 0.10 ± 0.30 for chlamydophilosis was observed. Small ruminant farmers in the Adamawa region showing a mean score of 0.04 ± 0.21 for toxoplasmosis and 0.04 ± 0.18 for chlamydophilosis were less sensitized than those in other regions (Figure 2(e)). When asked specifically about farmers' perception of the risks of toxoplasmosis and chlamydophilosis, 11% of farmers stated that these diseases were a threat to public health, but 10% were aware that they were a problem for animal health. Farmers in the Adamawa region (*P*=0.04) and respondents aged under 20 with primary education (0.04 ± 0.23) did not perceive toxoplasmosis and chlamydophilosis as a significant risk compared with other categories (Figure 6). However, the level of education (*P*=0.02), occupation (*P*=0.0001), purpose of farming (*P*=0.05), and feeding method (*P*=0.004) significantly influenced the risk perception of small ruminant farmers in the said localities (Table 2).

### 3.6. Association between KAPP Scores Determined for Small Ruminant Breeders in the Different Study Regions

Person's correlation was used to highlight the level of association between KAPPs by region. The associations showed that the KAPP scales of small ruminant farmers in the study regions were significantly (*P* < 0.01) and positively correlated between knowledge about toxoplasmosis and chlamydophilosis (*r* = 0.98) and risk perception (*r* = 0.99). Strong significant correlations were also observed between KAPP scales within the same region, ranging up to 0.99 between regions for toxoplasmosis knowledge, chlamydophilosis knowledge (*r* = 0.99), and risk perception (*r* = 0.99) (Table 3).

## 4. Discussion

The present study was conducted to assess the knowledge, attitudes, practices, and perception of zoonotic risks of toxoplasmosis and chlamydophilosis among small ruminant farmers in Cameroon. Although the majority of farmers interviewed (96.5%) had reported abortions in sheep and goats in the two years prior to the interview, the causes of abortions, their modes of transmission, preventive actions, and their risk to public health are rarely known and understood by these small ruminant farmers. The level of knowledge of toxoplasmosis and chlamydophilosis observed in the present study is lower than recorded previously in Ethiopia (22.4 ±33.6%) [24] and Zimbabwe (21.6%) [25] but similar to the knowledge scores obtained in northeastern China [26] although the latter surveyed poultry farmers. The lower knowledge score of the farmers in this study is associated with a higher proportion of respondents without education and lack of support from animal health professionals (96.7%). However, most of the respondents live in fairly remote rural areas under a high workload and therefore do not have access to information. Human behavior, education level, and communication between veterinarians and farmers play a major role in the control of toxoplasmosis and chlamydophilosis on farms [27]. Hence, the low level of knowledge among farmers about these diseases may also be the result of a lack of awareness among farmers and a lack of trained health education personnel [25, 28]. In the process of participatory animal disease control, a good knowledge of all links in the chain for better communication is important [29]. Based on the positive association between knowledge of chlamydophilosis and toxoplasmosis, training in agriculture and livestock is important for knowledge on abortive diseases, hygiene, and biosecurity measures in livestock. However, it is important to awareness of farmers by improving their knowledge on how to manage these diseases on the farm. The consumption of aborted animals by some farmers or the contamination of the environment by abortion products or cadavers demonstrates the lack of knowledge among Cameroonian farmers about the risks associated with improper management of abortion in livestock. However, an improvement in the legislation and regulations against clandestine breeding by certain breeders would improve the efficiency of breeding and access to veterinary services for this category of breeder. In this way, breeding constraints will be sought for better productivity but also to reduce the risk of transmission to humans of zoonotic pathologies located within the breeding farms. It is clear that poor livestock management creates a breeding ground for the expansion of these diseases and therefore responsible for human and animal infections in Cameroon [15, 16, 30, 31].

The undesirable attitudes toward the prevention of toxoplasmosis and chlamydophilosis from small ruminant birth products, such as assisting in the delivery and treatment of aborted animals with bare hands and improper disposal of aborted products (fetuses and placenta), confirm the need for appropriate health education to induce a change in attitude among small ruminant farmers toward the management of abortions and abortion products on their farms. It is important to sensitize farmers on the potential risk of zoonotic disease transmission by handling these abortion products. The undesirable attitude observed in the present study is lower than the proportions recorded in Ethiopia (37.3 ± 28.92%) [24]. A lack of correlation was noted between farmers' attitude, knowledge, practice, and perception of risk, which justifies the risky action of farmers in managing abortions on their farms. Farmers under 20 years of age whose main occupation is “rancher” and whose herd is greater than 30 heads behaved better than the other categories. In conclusion, education and behavioral change of farmers are of great importance in this participatory control.

The level of inappropriate practices in the present study was comparable to those obtained in Ethiopia [24, 32], Pakistan [33], and Tajikistan [34], although these studies focused on brucellosis. This practice among farmers in Cameroon was associated with marital status, occupation, presence of health personnel on the farm, and farms with more than 30 animals. With the objective of the farm being to generate financial income, farmers place less importance on their practice as long as they consider it profitable. Even though the majority (95%) of respondents has livestock as their primary occupation, it remains that education campaigns or increased support from veterinarians could enable farmers to be more efficient and vigilant about their actions on the farm. This is confirmed in this study by the fact that farmers who are monitored by animal health staff develop better behavioral practices in managing abortions on their farms. Rural veterinarians, who are closest to the farms in remote areas, are better positioned to expand community-based public health education and promotion activities to influence behavior change among farmers [35–37]. This is confirmed in this study by the fact that farmers who are monitored by animal health staff develop better behavioral practices in managing abortions on their farms. Rural veterinarians, who are closest to the farms in remote areas, are better positioned to expand community-based public health education and promotion activities to influence behavior change among farmers. This is why the authors cited above recommend, in the first instance, that the government veterinarians tasked with guiding health services must prioritize livestock health risks and allocate limited resources across disparate ecosystems with different disease threats. And the second instance, to identify livestock diseases of concern and strategies for improving herd health and resilience, we conducted community focus groups with pastoralists and interviewed pastoralist household leaders, village extension officers, and government veterinary officials. These approaches provide breeders with a better understanding of appropriate practices and attitudes. The involvement and participation of breeders in the management of abortions on their farms is an asset that contributes to the well-being of the community, especially in remote areas. It would therefore be important to set up a vigilance unit to give veterinarians in the field the means to make the right decisions. It is also important to involve farmers in this fight and to gain their confidence, and an improvement of the efficiency of their farms is necessary. To do this, biosecurity measures must be improved to prevent the entry of pathogens or their dissemination within the farms.

Despite the fact that the majority of the respondents had undesirable attitudes and inappropriate practices toward the prevention of toxoplasmosis and chlamydophilosis, nearly 12% of them, especially farmers with higher education level, aged over 71 years whose main function was financial livestock rearing and practicing zero grazing as a feeding method, significantly (*P* < 0.05) perceived these diseases as a risk to public health. This observation further demonstrates the importance of continuous education and awareness among farmers of all ages and education levels on the importance of improving their knowledge, adopting good attitude and practices in livestock farming to improve the profitability of this speculation [38].

## 5. Conclusion

This study assessed the knowledge, attitudes, practices, and risk perception of small ruminant farmers regarding toxoplasmosis and chlamydophilosis in Cameroon. The KAPP tool used revealed low overall knowledge of toxoplasmosis and chlamydophilosis, inadequate attitudes with inappropriate practices, and negative perceptions of risks related to abortion management on small ruminant farms. Breeding small ruminants in the Septentrion is an extensive activity, mainly carried out by men for financial gain, by young people, with an average herd size of 20 head. Herds are generally made up of two species (*C. aegagrus* and *O. aries*) and abortions are common in breeding. The study revealed low overall knowledge of toxoplasmosis and chlamydophilosis, inadequate attitudes, and inappropriate practices toward abortion management and negative risk perception of the farmers. Region, age, marital status, level of education, occupation of breeders, purpose of breeding as well as the level of hygiene on the farms, and the method of feeding were the main factors influencing the knowledge score, the attitudes, and practices. Respondents aged under 20 with a primary level of education did not perceive toxoplasmosis and chlamydia as a significant risk to human and animal health compared to other categories. The associations showed that the KAPP of small ruminant breeders in the regions studied were significantly (*P* < 0.01) and positively correlated between knowledge and risk perception. Significant correlations were also observed between KAPP scales within the same region and between regions. There is a need for targeted community health education programs to minimize the transmission of zoonotic pathogens from abortion products on small ruminant farms in Cameroon.

## Figures and Tables

**Table 1:** Distribution of mean knowledge scores on toxoplasmosis and chlamydophilosis according to demographic and farm characteristics of small ruminant farmers (*N* = 200) in the Adamawa, North, and Far North regions of Cameroon.

Factors	Variables	*N*	Toxoplasmosis	*P* value	Chlamydophilosis Mean ± SD	*P* value
			Mean ± SD			
Global knowledge		200	0.08 ± 0.23		0.07 ± 0.18	

Study regions	Adamawa	50	0.03 ± 0.14	0.05^*∗*^	0.03 ± 0.10	0.03^*∗*^
	North	81	0.12 ± 0.27		0.10 ± 0.21	
	Far North	69	0.07 ± 0.21		0.17 ± 0.10	

Sex	Male	160	0.07 ± 0.21	0.27	0.06 ± 0.16	0.23
	Female	40	0.12 ± 0.28		0.10 ± 0.23	

Age (year)	<20	4	0	0.001^*∗*^	0	0.001^*∗*^
	21–50	113	0.08 ± 0.22		0.07 ± 0.18	
	51–70	74	0.08 ± 0.23		0.06 ± 0.16	
	>71	9	0.17 ± 0.33		0.26 ± 0.26	

Level of education	No	73	0.11 ± 0.27	0.02^*∗*^	0.09 ± 0.21	0.27
	Primary	55	0.03 ± 0.13		0.03 ± 0.13	
	Secondary	55	0.07 ± 0.21		0.06 ± 0.15	
	Higher	17	0.14 ± 0.30		0.11 ± 0.22	

Marital status	Single	28	0.05 ± 0.20	0.001^*∗*^	0.05 ± 0.15	0.001^*∗*^
	Monogame	109	0.08 ± 0.22		0.07 ± 0.18	
	Polygamist	61	0.08 ± 0.22		0.06 ± 0.16	
	Widow	2	0.75 ± 0.07		0.60 ± 0.00	

Duration in the breeding (year)	<10	53	0.07 ± 0.22	0.81	0.06 ± 0.16	0.82
	11–30	127	0.08 ± 0.23		0.07 ± 0.18	
	>30	20	0.11 ± 0.27		0.09 ± 0.19	

Primary occupation	Village chief	2	0	0.05^*∗*^	0	0.05^*∗*^
	Trader	3	0		0	
	Student	4	0		0	
	Breeder	190	0.08 ± 0.23		0.07 ± 0.18	
	Public servant	1	0		0	

Breeding objectives	Financial	139	0.07 ± 0.22	0.05^*∗*^	0.06 ± 0.17	0.13
	Consumption	51	0.17 ± 0.31		0.13 ± 0.23	
	Pleasure	10	0.16 ± 0.34		0.12 ± 0.26	

Number of small ruminants in farms	<10	45	0.11 ± 0.25	0.63	0.08 ± 0.19	0.76
	10–30	131	0.07 ± 0.22		0.06 ± 0.17	
	>30	24	0.09 ± 024		0.07 ± 0.18	

Health personnel	Yes	7	0.30 ± 0.24	0.89	0.26 ± 0.22	0.91
	No	193	0.29 ± 0.24		0.26 ± 0.23	

Hygiene level	Clean	48	0.16 ± 0.32	0.03^*∗*^	0.14 ± 0.25	0.02^*∗*^
	Very clean	8	0.09 ± 0.25		0.06 ± 0.18	
	Dirty	134	0.06 ± 0.19		0.05 ± 0.14	
	Very dirty	10	0.01 ± 0.03		0.02 ± 0.04	

Power supply mode	Grazing	180	0.06 ± 0.21	0.001^*∗*^	0.06 ± 0.16	0.01^*∗*^
	Zero grazing	20	0.22 ± 0.33		0.16 ± 0.24	

Values in a column with “^*∗*^” differ significantly at *P* < 0.05; ANOVA results comparing mean factor scores by category while the independent *t*-test was used to compare factor scores of dichotomous variables; *N* = number of respondents; SD: standard deviation.

**Table 2:** Distribution of mean scores of practices, attitudes toward abortion management in small ruminants, and risk perception of toxoplasmosis and chlamydophilosis according to demographic and herder characteristics (*N* = 200) in Adamawa, North, and Far North regions of Cameroon.

Factor	Variables	*N*	Practices		Attitude		Perception of risk of toxoplasmosis and chlamydophilosis	
			Mean ± SD	*P* value	Mean ± SD	*P* value	Mean ± SD	*P* value
Study regions	Adamawa	50	0.38 ± 0.14	0.57	0.29 ± 0.07	0.01^*∗*^	0.04 ± 0.19	0.04^*∗*^
	North	81	0.36 ± 0.14		0.33 ± 0.07		0.18 ± 0.31	
	Extreme-North	69	0.36 ± 0.11		0.33 ± 0.08		0.10 ± 0.31	

Sex	Male	160	0.36 ± 0.13	0.71	0.32 ± 0.07	0.85	0.10 ± 0.32	0.34
	Female	40	0.37 ± 0.14		0.32 ± 0.08		0.16 ± 0.39	

Age in years	<20	4	0.44 ± 0.24	0.68	0.38 ± 0.05	0.05^*∗*^	0	0.05^*∗*^
	21–50	113	0.37 ± 0.14		0.32 ± 0.08		0.11 ± 0.33	
	51–70	74	0.36 ± 0.12		0.32 ± 0.07		0.11 ± 0.33	
	>71	9	0.31 ± 0.11		0.36 ± 0.09		0.25 ± 0.50	

Level of education	No	73	0.36 ± 0.12	0.28	0.32 ± 0.07	0.20	0.16 ± 0.39	0.02^*∗*^
	Primary	55	0.39 ± 0.14		0.34 ± 0.08		0.04 ± 0.22	
	Secondary	55	0.35 ± 0.14		0.32 ± 0.08		0.10 ± 0.31	
	Higher	17	0.33 ± 0.33		0.30 ± 0.06		0.19 ± 0.43	

Marital status	Single	28	0.34 ± 0.13	0.05^*∗*^	0.33 ± 0.06	0.89	0.08 ± 0.28	0.0001^*∗*^
	Monogame	109	0.36 ± 0.14		0.32 ± 0.08		0.11 ± 0.33	
	Polygamist	61	0.39 ± 0.11		0.32 ± 0.07		0.11 ± 0.32	
	Widow	2	0.25 ± 0.00		0.30 ± 0.14		1 ± 0.00	

Duration in the breeding (year)	<10	53	0.36 ± 0.15	0.91	0.32 ± 0.07	0.70	0.10 ± 0.31	0.79
	11–30	127	0.37 ± 0.12		0.32 ± 0.08		0.12 ± 0.34	
	>30	20	0.35 ± 0.13		0.31 ± 0.08		0.16 ± 0.38	

Primary occupation	Village chief	2	0.56 ± 0.09	0.05^*∗*^	0.35 ± 0.21	0.05^*∗*^	0	0.0001^*∗*^
	Trader	3	0.29 ± 0.08		0.30 ± 0.10		0	
	Student	4	0.41 ± 0.26		0.38 ± 0.05		0	
	Breeder	190	0.36 ± 0.13		0.32 ± 0.07		0.12 ± 0.34	
	Public servant	1	0.38 ± 0.00		0.20 ± 0.00		0	

Breeding objectives	Financial income	139	0.34 ± 0.10	0.45	0.34 ± 0.07	0.46	0.30 ± 0.51	0.05^*∗*^
	Consumption	51	0.31 ± 0.12		0.33 ± 0.08		0.25 ± 0.45	
	Pleasure	10	0.35 ± 0.10		0.35 ± 0.08		0.23 ± 0.48	

Number of small ruminants in farms	<10	45	0.41 ± 0.13	0.02^*∗*^	0.33 ± 0.07	0.05^*∗*^	0.15 ± 0.36	0.70
	10–30	131	0.35 ± 0.12		0.31 ± 0.07		0.10 ± 0.32	
	>30	24	0.36 ± 0.14		0.35 ± 0.08		0.13 ± 0.35	

Health personnel	Yes	7	0.59 ± 0.17	0.04^*∗*^	0.29 ± 0.30	0.68	0.37 ± 0.20	0.92
	No	193	0.55 ± 0.19		0.28 ± 0.30		0.37 ± 0.22	

Hygiene level	Own	48	0.35 ± 0.12	0.33	0.33 ± 0.09	0.57	0.23 ± 0.46	0.03
	Very clean	8	0.37 ± 0.13		0.33 ± 0.07		0.08 ± 0.28	
	Dirty	134	0.33 ± 0.15		0.32 ± 0.07		0	
	Very dirty	10	0.42 ± 0.11		0.35 ± 0.08		0.13 ± 0.35	

Power supply mode	Grazing	180	0.37 ± 0.13	0.52	0.32 ± 0.07	0.05^*∗*^	0.09 ± 0.30	0.004∗
	Zero grazing	20	0.35 ± 0.13		0.35 ± 0.08		0.32 ± 0.33	

Values in a column with “^*∗*^” differ significantly at *P* < 0.05; *N* = number of respondents; SD: standard deviation.

**Table 3:** Pearson correlation measures of small ruminant herders (*N* = 200) in the Adamawa, North, and Far North regions of Cameroon.

Parameters	Variable	Knowledge of chlamydophilosis	Knowledge of toxoplasmosis	Practices	Attitudes	*N*
Pooled	Knowledge of toxoplasmosis	0.98^*∗∗*^				200
	Practices	−0.09	−0.08			
	Attitudes	0.07	0.06	0.07		
	Risk perception	0.99^*∗∗*^	0.99^*∗∗*^	−0.08	0.07	

Adamawa	Knowledge of toxoplasmosis	0.99^*∗∗*^				50
	Practices	−0.15	−0.09			
	Attitudes	−0.06	−0.04	0.19		
	Risk perception	0.99^*∗∗*^	0.99^*∗∗*^	−0.10	−0.05	

North	Knowledge of toxoplasmosis	0.96^*∗∗*^				81
	Practices	−0.02	−0.04			
	Attitudes	0.13	0.14	0.14		
	Risk perception	0.99^*∗∗*^	0.99^*∗∗*^	−0.03	0.15	

Far north	Knowledge of toxoplasmosis	0.98				69
	Practices	−0.16	−0.13			
	Attitudes	−0.08	−0.14	−0.01		
	Risk perception	0.99^*∗∗*^	0.99^*∗∗*^	−0.13	−0.11	

Values in a column with “^*∗∗*^” differ significantly at *P* < 0.001; *N* = total number of respondents.

## Data Availability

The data used to support the findings of this study are included within the article.
